# Growth Hormone Secretagogue Receptor 1A Antagonist JMV2959 Effectively Prevents Morphine Memory Reconsolidation and Relapse

**DOI:** 10.3389/fphar.2021.718615

**Published:** 2021-11-29

**Authors:** Jing Zhao, Xinyu Du, Mingzhu Chen, Shimin Zhu

**Affiliations:** ^1^ Department of Anesthesiology, Shanghai Jiao Tong University Affiliated Sixth People’s Hospital, Shanghai, China; ^2^ Key Laboratory of Combinatorial Biosynthesis and Drug Discovery (Wuhan University), Ministry of Education, and Wuhan University School of Pharmaceutical Sciences, Wuhan, China

**Keywords:** conditioned place preference, morphine relapse, addiction, ghrelin antagonism, JMV2959

## Abstract

Relapse to drug seeking after prolonged abstinence is a major problem in the clinical treatment of drug addiction. The use of pharmacological interventions to disrupt established drug reward memories is a promising strategy for the treatment of drug addiction. A growth hormone secretagogue receptor 1 A antagonist, JMV2959, has been shown to reduce morphine-induced conditioned place preference (CPP) in rats within hours of intervention; thus, JMV2959 is a potential candidate for drug addiction treatment. However, the effect of JMV2959 on reconsolidation to disrupt drug seeking remains unknown. In this study, we assessed the effect of JMV2959 on morphine induced memory reconsolidation to inhibit drug seeking after drug withdrawal. Our results showed that the administration of JMV2959 (6 mg/kg) significantly reduced environmental cue induced CPP, which suggested a preventive effect of JMV2959 on morphine induced memory reconsolidation. Additionally, JMV2959 administration significantly altered the locomotor activity and food and water intake but did not significantly alter the natural reward preference. We concluded that JMV2959 may be an effective candidate to treat drug addiction.

## Introduction

Drug addiction is a chronic relapsing brain disorder that is often caused by the persistent intake of morphine, cocaine, amphetamine, or fentanyl over a long period (Leshner, 1997; O'Brien and McLellan, 1996). These addictive drugs activate human neurological systems through neurotrophic factors to form drug reward memory. This newly learned memory can be transformed into stable memories *via* memory consolidation (Davis and Squire, 1984; McGaugh, 2000). When this stabilised drug memory is recalled or reactivated, it undergoes additional consolidation, known as reconsolidation. This process requires *de novo* protein synthesis mediated by receptors, signal transduction pathways, and proteins. The reconsolidated drug reward memory is labile and sensitive to certain disruptors, such as receptor antagonists, pharmacological techniques, and protein synthesis inhibitors (Sara, 2000; Nestler, 2001; Hyman et al., 2006; Tronson et al., 2006; Tronson and Taylor, 2007). Therefore, the disruption of drug reward reconsolidation has become a promising therapeutic strategy for the prevention of drug addiction. Protein synthesis inhibitors, such as anisomycin or cycloheximide, administered immediately after reactivation, can disrupt drug reward memory, which leads to the absence of addictive behaviour (Milekic et al., 2006; Valjent et al., 2006; Robinson and Franklin, 2007). However, during drug addiction treatment, relapse to drug seeking after prolonged abstinence is a major clinical problem (Grimm et al., 2001; Lu et al., 2005; Li et al., 2008a; Millan et al., 2013). Thus, new effective treatment strategies are of urgent need.

Ghrelin, a 28-amino acid peptide hormone endogenously expressed in the gut and brain tissues, plays a crucial role in food and addictive drug rewards by binding to and activating the growth hormone secretagogue receptor (GHSR1A) (Wu et al., 2019; Abizaid and Hougland, 2020). The activation of the ghrelin-GHSR1A signalling pathway is critical to adjust appetite and food intake in the gut (Kern et al., 2015). The ghrelin and GHSR1A expressed in areas of the brain, such as the hypothalamus, striatum, nucleus accumbens, amygdala, prefrontal cortex, hippocampus, and ventral tegmental area, have been reported to play a crucial role in drug intake and reward (Howard et al., 1996; Abizaid et al., 2006; Ferrini et al., 2009; Landgren et al., 2011; Skibicka et al., 2011).

Recently, GHSR1A antagonism has been adopted as a promising anti-addiction mechanism. Studies have demonstrated that the administration of GHSR1A antagonist JMV2959 significantly inhibits fentanyl‐ and methamphetamine-induced conditioned place preference (CPP), intravenous self-administration, and dopamine release in the nucleus accumbens in rats (Engel et al., 2015; Jerabek et al., 2017; Havlickova et al., 2018; Sustkova-Fiserova et al., 2019). However, the effect of JMV2959 on relapse to drug seeking remains unknown. In this study, we examined the effect of JMV2959 on the reconsolidation of drug reward memory and prevention of relapse to drug seeking in a morphine-induced CPP animal model. We also examined the effect of JMV2959 on natural reward preference and food and water intake.

## Materials and Methods

### Animals

Male Sprague–Dawley rats (weighing 220–250 g) were housed in a temperature (23 ± 2°C) and humidity (50 ± 5%) controlled animal facility. A total of 48 rats were randomized into experimental groups with free access to food and water (24 rats for CPP, 12 rats for natural reward preference, 12 rats for food, water intake and locomoter activity). Rats weighed 300–320 g when the experiments began. The experimental procedures were performed in accordance with the National Institutes of Health Guide for the Care and Use of Laboratory Animals and were approved by the Wuhan University Animal Care and Use Committee (Li et al., 2011; Lin et al., 2014).

### Drugs

JMV2959 (1,2,4-triazole derivate, purity >99.5%) was chemically synthesised by Waterfall Biotechnologies, LLC, Shanghai, China. The compound was dissolved in 2% dimethyl sulphoxide at different concentrations for subcutaneous and intraperitoneal injections. Morphine sulphate (Qinghai Pharmaceutical Ltd., Xining, China) was prepared at different concentrations in 0.9% physiological saline for subcutaneous and intraperitoneal injection (Li et al., 2011; Lin et al., 2014). Sucrose (2%) solution was dissolved in tap water.

### CPP: Effect of JMV2959 on Morphine Reward Memory Reconsolidation and Relapse to Morphine

CPP training was performed using an unbiased, counterbalanced protocol as described in the previous studies (Li et al., 2008a; Li et al., 2008b). Briefly, the CPP apparatus was assembled by five identical three-chamber polyvinyl chloride (PVC) boxes. In each box, two large side chambers (27.9 cm long, 21.0 cm wide and 20.9 cm high) were separated by a smaller one (12.1 cm long, 21.0 cm wide and 20.9 cm high with a smooth PVC floor). The two larger chambers differed in their floor texture (bar or grid) and provided distinct visual contexts that were paired with the drug or saline injection. Guillotine doors were manually installed to separate the three distinct chambers.

To determine the baseline preference, rats were initially placed in the middle chamber with the doors removed for 15 min (pre-conditioning test). A computer measured the time spent in the designated saline- or morphine-paired chambers during the 15 min session by the interruption of infrared beams by animals. The data (not shown) indicated that most rats spent approximately one-third of the time in each chamber. Approximately 5% of rats exhibited a strong unconditioned preference (540 s) and were excluded from the study.

Rats (*n* = 6 for each group) were assigned to one of the following treatments: *1. saline CPP + saline (with 2% dimethyl sulphoxide) (S + S)*; 2. saline CPP + JMV2959 (S + J); *3. morphine CPP + saline (with 2% dimethyl sulphoxide) (M + S)*; 4. morphine CPP + JMV2959 (S + J). On the conditioning days, each rat was trained for eight consecutive days with alternate injections of morphine (10 mg/kg, s.c., on day 2, 4, 6, and 8) or vehicle (morphine control, 1 ml/kg, s.c., on day 3, 5, 7, and 9). After each injection, rats were confined to the corresponding conditioning chambers for 45 min before returning to their home cages. The day after conditioning (day 10), rats were tested for CPP (post-conditioning test) under conditions identical to those described in the pre-conditioning test. The CPP score was defined as the time spent, in seconds, in the morphine-paired chamber minus that spent in the morphine-unpaired (saline-paired) chamber (Lin et al., 2014).

#### Drug-Memory Reactivation

Twenty-four hours later (day 11), rats were exposed to the morphine-paired chamber for 10 min immediately followed by JMV2959 (3, 6 mg/kg,i.p) or vehicle administration. Two groups of rats (*n* = 6 per group) were used.

#### Retesting and Priming of Morphine CPP

The drug-induced CPP was retested 1 day (post-treatment 1, Post-T1) or 7 days (post-treatment 7, Post-T7) after memory reactivation (day 12 and 18, respectively). If rats did not show significant drug CPP, they would be injected with a priming drug injection (3 mg/kg morphine, s.c.) and immediately submitted to the CPP procedures again (post-treatment 8, Post-T8).

### Locomotor Activity

The behavioural locomotor activity test was performed based on the previous studies (Li et al., 2008a; Li et al., 2011; Lin et al., 2014). Briefly, the rats were placed in a photocell cage for 1 h according to the behavioural testing procedure. Then, they were injected with vehicle or JMV2959 (6 mg/kg JMV2959, i.p., respectively) and immediately placed in the chamber. Ambulation behaviour was measured for 60 min. After each trial, the chamber was carefully cleaned.

### Natural Reward Preference and Food and Water Intake

To test the effect of JMV2959 on natural reward preference, the two-bottle sucrose intake test was performed as described in the previous studies (Li et al., 2008a; Li et al., 2011; Lin et al., 2014). Rats were housed singly during the testing. The procedure included: 1) Adaptation: On day 1, a bottle filled with tap water and a bottle filled with 2% sucrose solution were placed in symmetric positions on the same cage wall at 18:00 h. On day 2, the placements of the water and sucrose bottles were switched to balance side preference; 2) Grouping: The rats were divided into two groups (JMV2959 0 or 6 mg/kg) fairly according to their weight; 3) Testing: Rats were administered JMV2959 injections (0 or 6 mg/kg) on day 3 in the morning. The results were measured 24 h later.

The rats.were provided with a known quantity of water in the nostled drinking bottle, which was replenished every morning after recording daily consumption. For food intake assay, a known quantity of food were provided and intake was calculated by the quantity difference after daily consumption. The animals were weighed and their weight was recorded in grams.

### Statistics

Data are expressed as mean ± S.E.M. The between-factor differences for JMV2959 treatment doses (0, 3, 6 mg/kg) and the within-factor differences for the test condition (pre- and post-conditioning and post-treatment) were analysed using a two-way repeated-measures analysis of variance (ANOVA). For locomotor activity, a two-way repeated-measures ANOVA was used to analyse the differences in crossings. The rest of the experiments were analysed with a two-way ANOVA. Behavioural data were analysed using SPSS 15.0. Differences were statistically significant when *p* < 0.05.

## Results

### JMV2959 Disrupts the Reconsolidation of Morphine Reward Memory

**FIGURE 1:**
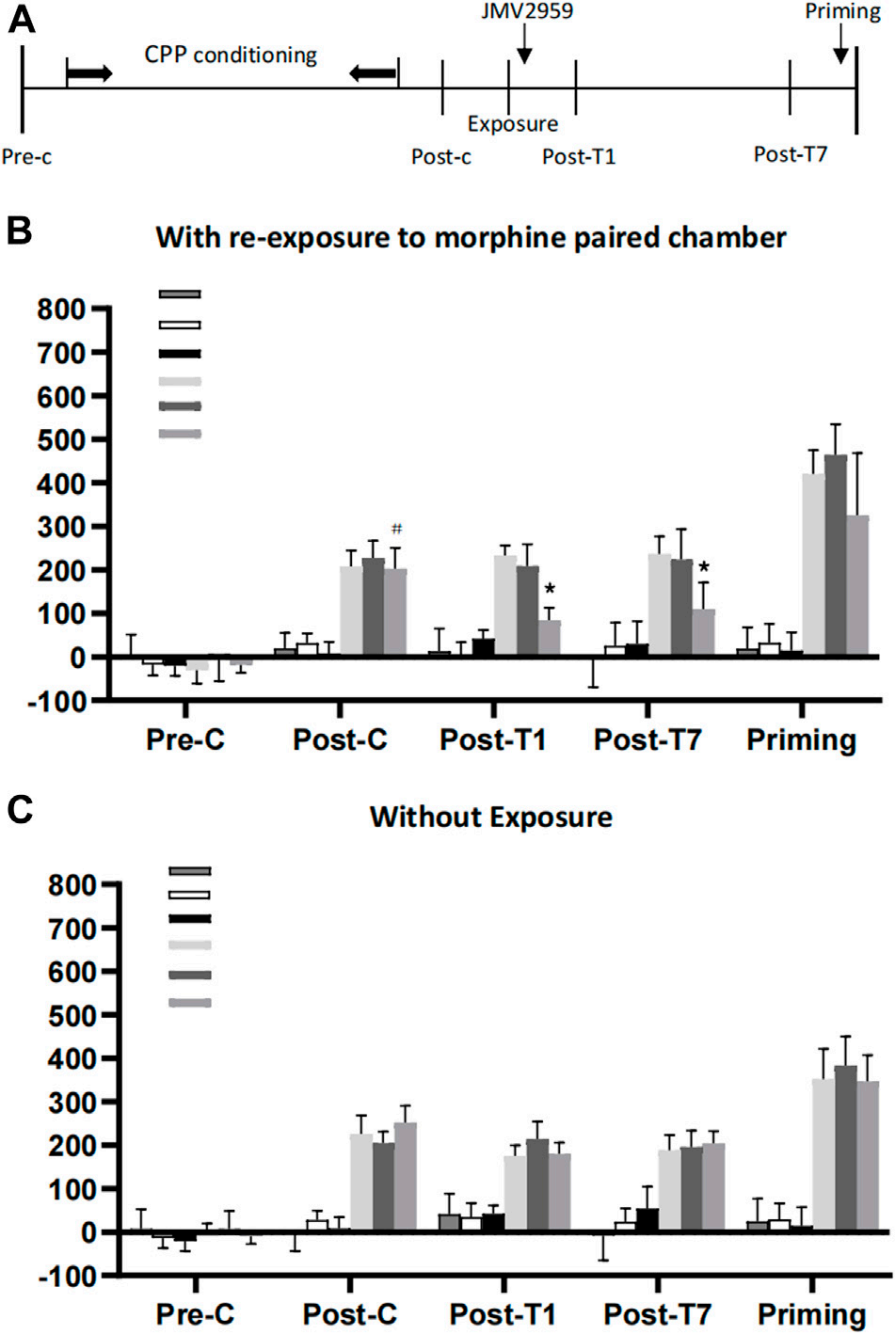
The effect of JMV2959 for rats on morphine reconsolidation. **(A)** Timeline of the Experimental procedure **(B)** Systematic administration of JMV2959 immediately after exposure to morphine-paired context impaired the reconsolidation of morphine reward memory. The inhibitory effect of JMV2959 on the expression of morphine CPP last for 7d. When given 3 mg/kg morphine priming injection, rats in the group with 6 mg/kg JMV2959 treatment immediately after exposure reinstated the morphine CPP (*n* = 6-8 per group). *p < 0.01 *vs*. preconditioning or post-conditioning within group. #*p* < 0.01 *vs*. preconditioning or post-treatment within group. **(C)** Systemic administration of JMV2959 without exposure to morphine reward memory. Pre-C, preconditioning; Post-C, post-conditioning; Post-T1, post-treatment; Post-T14, post-treatment 14; Priming, injected by 3 mg/kg morphine.

In experiment 1, a three-way ANOVA conducted on CPP score using exposure (exposure to drug-paired or no exposure) and JMV2959 (0, 3 or 6 mg/kg) as the between-subjects factors and test condition (pre-conditioning, post-conditioning and post-treatment 1 or 7) as the within-subjects factor, revealed that there was a signifificant interaction between exposure × JMV2959 × test condition(F1,287 = 18.19; *p* < 0.01) and exposure ×JMV2959(F_(3,189)_ = 45.43; *p* < 0.01) and exposure × JMV2959(F_(3,189)_ = 34.23; *p* < 0.01). Post-hoc analysis showed that after morphine CPP training, all groups acquired CPP (*p* < 0.001) and there were no differences in CPP scores between any two groups during post-conditioning. Compared with the post-conditioning test, the CPP score was signifificantly decreased only in the group of rats administrated a dose of 6 mg/kg but not 1 mg/kg JMV2959 after re-exposure to the previously morphinepaired chamber (*p* < 0.01) on the post-treatment day 1 or 7, as shown in (Figures 1A,B). Thus, the inhibitory effect of JMV2959 on reconsolidation of morphine reward memory was dependent on re-exposure to drug-paired context and memory reactivation in a dose-dependent way (Figure 1B). And this absence of CPP was reinstated by a morphine priming injection (one-way repeated measures ANOVA showed F1,168 = 65.69; *p* < 0.05, compared to post-treatment condition) (Figure 1C).

### JMV2959 has an Effect on Locomotor Activity

**FIGURE 2:**
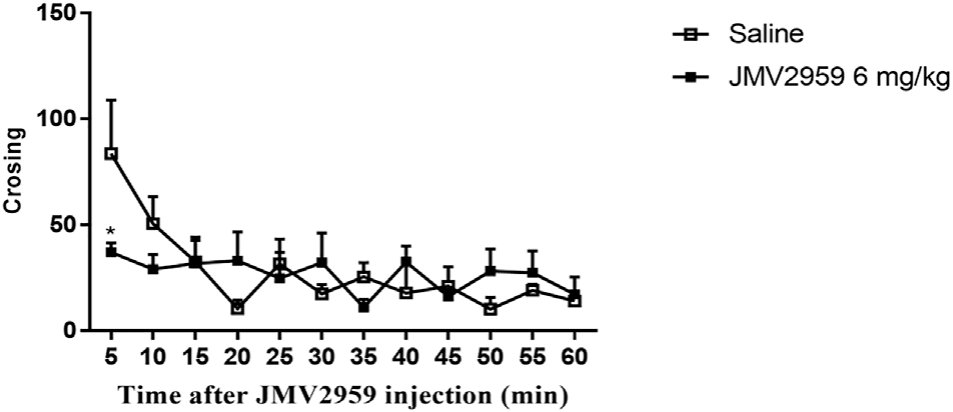
The effect of JMV2959 for rats on locomotor activity. The rats were placed in a photocell cage for 1 h according to the behavioural testing procedure. Then they were injected with vehicle or JMV2959 (0 or 6 mg/kg JMV2959, i.p., respectively) and immediately placed in the chamber. Ambulation behaviour was measured for 60 min. Values are presented as the mean ± SEM (*n* = 6) *p < 0.05, **p < 0.01.

Locomotor activity was detected to explore whether JMV2959 affects rat locomotion, which could influence the CPP score. Two-way repeated-measures ANOVA analyse indicated that there was significant difference between the group exposed to JMV2959 (6 mg/kg) and the saline group(ANOVA, F_(1,15)_ = 45.46, *p* < 0.01), which indicated that JMV2959 was able to influence the locomotor activity of the rats and that the CPP score was valid (Figure 2).

### JMV2959 May Have Weak Effect on Natural Reward Preference

**FIGURE 3:**
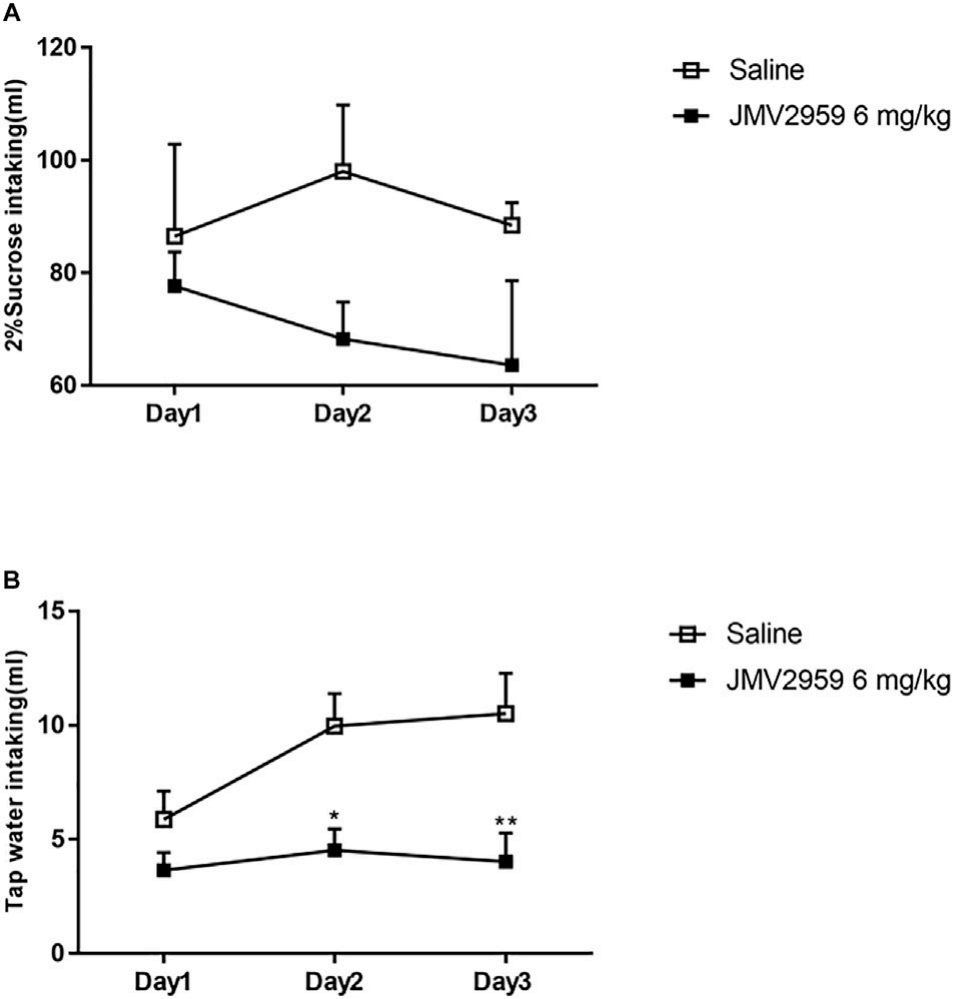
Effect of JMV2959 on natural reward preference. The two-bottle sucrose intake test was performed as described in methods. The difference between Saline group (*n* = 6) and the group with JMV2959 (6 mg/kg, *n* = 6) were measured by **(A)** 2% sucrose and **(B)** tap water intaking up to 3 days. Two-way repeated measures ANOVA was taken. Values are presented as the mean ± SEM (*n* = 4). *p < 0.05, **p < 0.01.

The two-bottle sucrose intake test was conducted to determine the effect of JMV2959 on natural reward preference. There was no significant difference between the rats with exposure to JMV2959 and those without(F_(1,39)_ = 0.54, *p* > 0.05). However, the trend suggests that JMV2959 may have weak influence on natural reward preference and there could be a significant difference should larger sample number be included (Figure 3A). Water intake was recorded at the same time and the JMV2959-injected rats showed significantly less water drinking on the second (*p* < 0.05) and third (*p* < 0.01) days than the non-JMV2959-injected rats (Figure 3B).

### JMV2959 Causes Less Food Intake and Weight Loss

**FIGURE 4:**
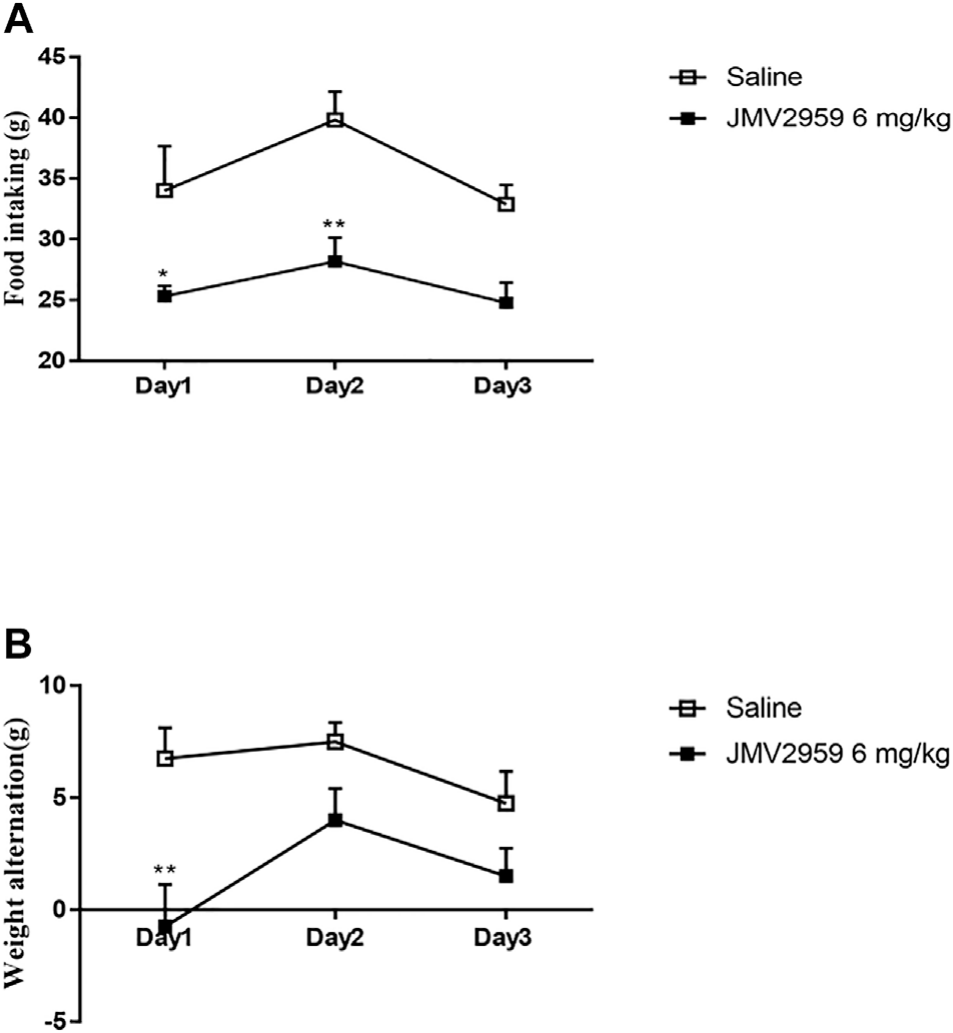
Effect of JMV2959 on food intake and body weight. Weight alteration and food intake test were measured as described in methods. The difference between saline group (*n* = 6) and the group with JMV2959 (6 mg/kg, *n* = 6) were recorded by **(A)** food intaking and **(B)** body weight in 3 days. Two-way repeated measures ANOVA was taken. Values are presented as the mean ± SEM (*n* = 4).*p < 0.05,**p < 0.01.

Weight alteration and food intake were recorded because JMV2959 is a GHSR1A antagonist that plays a crucial role in food intake. The JMV2959-injected rats showed significantly less food intake than the saline group on the firstand second days (ANOVA, F_(1,39)_ = 34.3, *p* < 0.05) (Figure 4A). The weight alteration in the JMV2959-injected group was significantly less than that in the saline group on the first day (ANOVA, F_(1,39)_ = 59.2, *p* < 0.01) (Figure 4B).

## Discussion

Drug addiction is a life-threatening disease and huge medical burden; however, the prevention of drug abuse is a largely unmet need. In this study, we showed the preventive effect of the GHSR1A antagonist JMV2959 on morphine memory reconsolidation and relapse to morphine over a long period. Our results are consistent with those of a previous study that suggested the inhibitory effect of JMV2959 on morphine-induced CPP(Engel et al., 2015; Jerabek et al., 2017). We also showed that the systemic administration of JMV2959 was long-lasting and effective, which suggests a crucial role of the ghrelin-GHSR1A pathway in the regulation of drug addiction. Other studies have reported the inhibitory effect of JMV2959 on addictive drug reward memory in other drugs, such as methamphetamine, fentanyl, and cocaine (Engel et al., 2015; Jerabek et al., 2017; Havlickova et al., 2018; Sustkova-Fiserova et al., 2019).

Drug seeking is a major problem in the treatment of drug addiction that is often caused by maladaptive drug-related memory (Lin et al., 2014). The prevention of relapse is a huge challenge in clinical practice owing to drug-seeking behaviours induced by drug-associated environmental cues (Li et al., 2011; Lin et al., 2014). The disruption of drug memory reconsolidation has been suggested as a promising strategy to prevent relapse (Lin et al., 2014). A number of studies have demonstrated that ghrelin and its specific receptor GHSR1A are involved in the mediation of memory reward-related neurological processes (Havlickova et al., 2018; Sustkova-Fiserova et al., 2019). Thus, targeting these neuronal processes could disrupt memories underlying addiction behaviour (Engel et al., 2015; Jerabek et al., 2017). In this study, we employed JMV2959 to antagonise GHSR1A-mediated processes to disrupt reconsolidation to prevent drug seeking.

Our result presented an effective method to prevent drug seeking by the systemic administration of JMV2959, which has also been suggested by Engel group and Jerabek group, etc (Engel et al., 2015; Jerabek et al., 2017). Other studies have reported that rapamycin may be effective in rats (Lin et al., 2014). As rapamycin inhibits the mammalian target of rapamycin signaling pathway to disrupt protein synthesis that is dependent on long-term synaptic plasticity and memory storage (Casadio et al., 1999; Stoica et al., 2011), a ghrelin-GHSR1A antagonism mechanism could be a promising strategy to prevent drug addiction. Ghrelin-GHSR1A signaling has been reported to play a crucial role in drug intake and reward (Ge et al., 2018). Thus, it would be useful to design and synthesise more potent antagonists than JMV2959 to prevent drug addiction.

Ghrelin exerts a complex spectrum of effects on systemic metabolism, such as the stimulation of gut motility and gastric acid secretion, regulation of glucose metabolism, inhibition of insulin secretion, and increase of adiposity. Furthermore, it has effects on drug addiction and manipulates sleep, stress, and anxiety. In this study, JMV2959 significantly decreased water and food intake, which suggests that ghrelin-GHSR1A antagonism may also play a role in these behavioural differences.

## Conclusion

In this study, we elucidated that JMV2959 was able to inhibit morphine memory reconsolidation and relapse to drug seeking, suggesting that JMV2959 may be an effective candidate to treat drug addiction. Secondly, JMV2959 administration significantly affected the food and water consumption. Thirdly, JMV2959 may have an effect on altering the locomotor activity or natural reward preference.

## Data Availability

The original contributions presented in the study are included in the article/Supplementary material, further inquiries can be directed to the corresponding author.

## Abbreviations

CPP, conditioned place preference; GHSR1A, growth hormone secretagogue receptor.
